# Gut Microbiota in Psychiatric and Neurological Disorders: Current Insights and Therapeutic Implications

**DOI:** 10.3390/biomedicines13092104

**Published:** 2025-08-29

**Authors:** Marta Dziedziak, Agata Mytych, Hubert Paweł Szyller, Maria Lasocka, Gabriela Augustynowicz, Joanna Szydziak, Aleksandra Hrapkowicz, Maciej Dyda, Joanna Braksator, Tomasz Pytrus

**Affiliations:** 1 Student Scientific Group of Pediatric Gastroenterology and Nutrition, Wroclaw Medical University, 50-369 Wroclaw, Poland; marta.dziedziak@student.umw.edu.pl (M.D.); agata.mytych@student.umw.edu.pl (A.M.); marysialasocka@gmail.com (M.L.); gabriela.augustynowicz@interia.pl (G.A.); joannaszydziak1@gmail.com (J.S.); aleksandra.d.hrapkowicz@gmail.com (A.H.); macdyda@gmail.com (M.D.); 2 2nd Clinical Department of Paediatrics, Gastroenterology and Nutrition, Wroclaw Medical University, 50-369 Wrocalw, Poland; joanna.braksator@umw.edu.pl (J.B.); tomasz.pytrus@umw.edu.pl (T.P.)

**Keywords:** microbiota, major depressive disorder, schizophrenia, bipolar disorder, autism spectrum disorder, attention-deficit hyperactivity disorder, gut dysbiosis, neurodegenerative disorders, gut–brain axis

## Abstract

Recent studies increasingly highlight the complex interaction between gut microbiota and mental health, drawing attention to the role of the microbiota–gut–brain axis (MGBA) in the pathophysiology of mental and neurodevelopmental disorders. Changes in the composition of the gut microbiota—dysbiosis—are associated with conditions such as depression, schizophrenia, bipolar disorder (BD), autism spectrum disorders (ASD), attention deficit hyperactivity disorder (ADHD), and neurodegenerative diseases such as Parkinson’s and Alzheimer’s. These microbial imbalances can affect brain function through a variety of mechanisms, including activation of the immune system, alteration of intestinal permeability, modulation of the digestive and central nervous systems, and changes in the production of neuroactive metabolites such as short-chain fatty acids, serotonin, and tryptophan derivatives. The aim of this paper is to review the current state of knowledge on therapeutic strategies targeting the gut microbiome—including probiotics, prebiotics, synbiotics, personalized dietary interventions, and fecal microbiota transplantation (FMT)—which are becoming promising adjuncts or alternatives to conventional psychopharmacology, offering a forward-looking and individualized approach to mental health treatment. Understanding the bidirectional and multifactorial nature of MGBA may pave the way for new, integrative treatment paradigms in psychiatry and neurology, requiring further research and exploration of their scope of application.

## 1. Introduction

Mental health is increasingly recognized as a critical component of overall well-being, with psychiatric disorders representing a growing public health concern worldwide. According to the World Health Organization (WHO), mental illnesses account for approximately one in every six years lived with disability and are associated with a reduction in life expectancy of 10 to 20 years among individuals with severe mental disorders [1]. While pharmacotherapy and psychotherapy remain the primary treatments for psychiatric conditions, there is a pressing need for adjunctive or alternative strategies that may be associated with fewer adverse effects.

This need has driven growing interest in underexplored therapeutic targets, among which the gut microbiota has emerged as particularly promising. The human body harbors over 100 trillion microorganisms—outnumbering human cells by a factor of ten. The intestinal microbiota of a healthy adult weighs about 1 kg and comprises bacteria, viruses, protozoa, fungi, and archaea [2]. These microbial communities are essential not only for digestion and nutrient absorption, but also for immune regulation, vitamin synthesis, and pathogen defense, playing a key role in maintaining physiological homeostasis [2].

Their influence on mental health is largely mediated through the gut–brain axis—a bidirectional communication system linking the gastrointestinal tract and the central nervous system. Disruptions in the gut microbiota have been implicated in several psychiatric conditions, including depression, anxiety disorders, autism spectrum disorder, and schizophrenia [3,4]. Both clinical and experimental evidence suggest that modulation of the microbiome—through diet, probiotics, or other interventions—may offer novel avenues for improving mental health outcomes [5,6].

In addition to primary psychiatric disorders, alterations in the gut microbiota have also been associated with neurodegenerative diseases such as Parkinson’s and Alzheimer’s. Although these conditions are not classified as psychiatric illnesses per se, they frequently present with psychiatric symptoms such as depression, anxiety, and apathy. As such, their inclusion in microbiome-related research is both relevant and valuable [7].

The literature included in the review was searched in databases with a focus on clinical trials and original articles, followed by meta-analyses then literature reviews. The time range of papers recruited for the review assumed a limit of 10 years, of which some articles of special or historical interest were included despite exceeding the limit set. The keywords used in the research are listed above. Articles not directly related to the topic, not available in English, and outdated articles were not included. With the above restrictions, the authors selected 139 articles.

**Figure 1:**
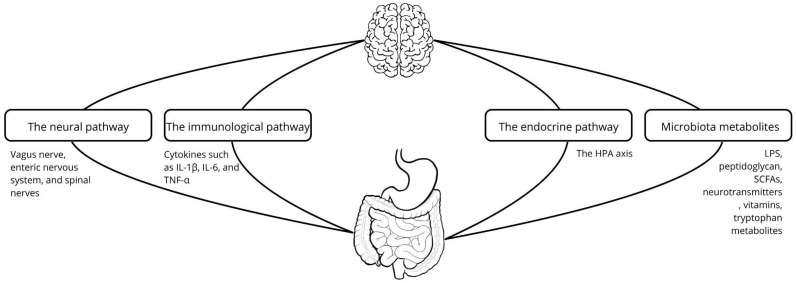
Schematic representation of the interrelationships on the gut–brain axis. The gut–brain axis is mediated through multiple direct and indirect pathways. These pathways include: (1) neural routes involving the enteric nervous system (ENS), vagus nerve, and spinal nerves; (2) neuroendocrine signaling, primarily via the hypothalamic–pituitary–adrenal (HPA) axis; (3) immune mechanisms involving cytokines such as IL-1β, IL-6, and TNF-α; and (4) microbiota-derived metabolites and neuroactive compounds, including short-chain fatty acids (SCFAs), neurotransmitters, vitamins, and tryptophan metabolites [2,8,9,10,11].

This paper provides a comprehensive review of current literature on the relationship between gut microbiota and psychiatric disorders. It also briefly addresses selected neurodegenerative diseases where psychiatric symptoms are prominent, offering a broader context and highlighting potential directions for future research in this rapidly evolving field. Schematic representation of the interrelationships on the gut–brain axis is presented on Figure 1.

## 2. Gut Microbiota—Essential Facts

The human gut microbiota provides numerous benefits to its host by performing essential physiological functions, including maintaining gut barrier integrity, influencing epithelial homeostasis, defending against pathogens, harvesting energy, and regulating both the immune system and the host’s brain processes and behavior [12,13]. It is primarily composed of *Proteobacteria*, *Firmicutes*, *Actinobacteria*, and *Bacteroidetes* [8,9]. It evolves throughout life, influenced by factors such as type of birth, diet, lifestyle, infections, medical conditions, and treatments [4,14,15,16,17]. Vaginally delivered infants acquire a microbiota resembling their mother’s vaginal and fecal flora, rich in *Lactobacillus* spp., while C-section infants develop a skin-like microbiota dominated by *Staphylococcus*, *Corynebacterium*, and *Propionibacterium* spp. Later on, its diversification is largely shaped by nutritional factors. Breastfed infants exhibit an increased abundance of certain *Bifidobacterium* species, which diversifies after weaning, whereas formula-fed infants typically have a lower prevalence of Bifidobacteria and a higher abundance of coliforms, *Bacteroides*, and *Clostridium difficile* [2,10]. When talking about the digestive tract we cannot disregard its first part—the oral cavity. This area is predominated by bacteria such as *Streptococci*, *Gemella*, *Granulicatella* and *Veillonella* [18], however some diseases such as periodontitis can cause a dysbiosis and progressive shift of dominance to Gram-negative species. The perfect example is *Porphyromonas gingivalis*, a Gram-negative, anaerobic bacterium which destroys gum tissue and is specifically linked to Alzheimer’s disease [19,20]. A narrative review of the studies published in recent years, written by Seyedmoalemi, emphasizes not only the bidirectional relationship between periodontitis and Alzheimer’s disease, but also the influence of oral health on the cognitive decline. Results show that periodontitis significantly increase the risk of dementia and mortality, particularly in individuals over 65 years of age. Another study shows, that oral dysbiosis can leads not only to Alzheimer disease, but also Parkinson’s disease and possibly Lewy- Body disease. Oral dysbiosis can potentially contribute, to even worsening the progression in multiple sclerosis, due to its role in systemic inflammation [18,19,20]. Long-term gut microbial composition remains susceptible to external influences such as dietary patterns, medications, pollutants, and psychosocial stressors. Diets rich in plant-based foods and micronutrients like magnesium are associated with reduced pro-inflammatory taxa (e.g., *E. coli*, *Clostridium innocuum*) and increased beneficial anaerobes (e.g., *Faecalibacterium prausnitzii*, *Agathobaculum butyriciproducens*). Conversely, diets high in animal-derived products favor bile-tolerant microorganisms, including *Alistipes*, *Bilophila*, and *Bacteroides* [17,21].

Probiotics have shown considerable potential in modulating the gut microbiota and supporting both physical and mental health. Lactobacillus strains present in fermented foods—such as kimchi, sauerkraut, and vinegar—have been associated with a range of health-promoting effects, including enhanced immune function and easing constipation [22]. A study by Tillisch et al. (2013) demonstrated that a four-week intake of a fermented milk product containing *Bifidobacterium animalis* subsp. *lactis*, *Lactococcus lactis* subsp. *lactis*, *Lactobacillus bulgaricus*, and *Streptococcus thermophilus* altered activity in brain regions involved in the central processing of emotion and sensation [23]. Building on these findings, the use of engineered probiotics has emerged as a promising approach for targeted therapeutic delivery, particularly in immunotherapy and vaccine development. Early efforts concentrated on intestinal disorders such as inflammatory bowel disease (IBD), where genetically modified *Lactococcus lactis* was engineered to produce the anti-inflammatory cytokine interleukin-10 (IL-10) in the gut. These applications demonstrated the capacity of engineered microbes to modulate immune responses and reduce local inflammation [24,25].

**Figure 2:**
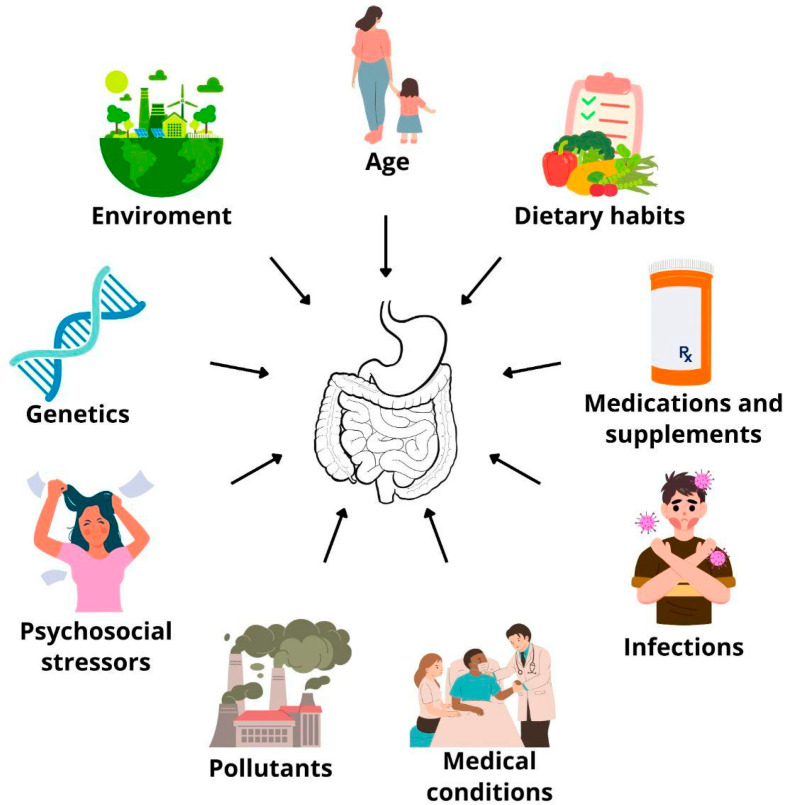
Selected factors and their impact on the gut microbiota. The composition of the gut microbiota is influenced by a range of factors throughout the lifespan, including dietary patterns (e.g., Western vs. Mediterranean diets), use of medications and supplements (such as probiotics), underlying medical conditions, exposure to environmental pollutants, psychosocial stress, host genetics, and environmental variables such as urban versus rural living and geographical location [2,8,9,17,22].

Alterations in gut microbiota composition have also been implicated in various gastrointestinal disorders. In patients with Crohn’s disease (CD), a notable decrease in beneficial anti-inflammatory bacteria such as *Firmicutes*, *Bacteroidetes*, *Akkermansia muciniphila*, and *Faecalibacterium prausnitzii* has been observed, alongside an increase in pro-inflammatory taxa including *Actinobacteria* and *Proteobacteria* [26]. In irritable bowel syndrome (IBS), alterations include elevated levels of *Firmicutes*, reduced levels of *Bacteroidetes*, and an increased *Firmicutes:Bacteroidetes* ratio at the phylum level. At lower taxonomic levels, elevated abundance of *Clostridia* and *Clostridiales*, alongside a reduction in *Bacteroidia* and *Bacteroidales*, was observed [27]. IBS often co-occurs with psychiatric conditions such as depression and anxiety, a pattern also observed in small intestinal bacterial overgrowth (SIBO), which is characterized by excessive bacterial growth in the small intestine [28,29]. Chojnacki et al. (2022) reported altered tryptophan metabolism in depressive patients with SIBO, showing increased urinary kynurenine and quinolinic acid levels alongside decreased tryptophan and kynurenic acid, suggesting enhanced activation of the neurotoxic kynurenine pathway [30]. Additionally, SIBO patients scored higher on depression and anxiety scales (Hamilton Depression Rating Scale (HAM-D) and the Hamilton Anxiety Rating Scale) compared to controls [31]. These findings may suggest a link between gastrointestinal disorders, dysbiosis, and the development of neuropsychiatric and neurodegenerative conditions [29,32]. Selected factors affecting the composition of the human gut microbiota are summarized in Figure 2.

## 3. Brain–Gut Axis

The gut–brain axis (GBA) is a bidirectional communication system linking the central nervous system (CNS) with the enteric nervous system (ENS). The CNS interacts with the gut microbiota through several direct and indirect pathways, including endocrine signaling—primarily via the hypothalamic–pituitary–adrenal (HPA) axis, immune responses mediated by cytokines, metabolic routes involving neurotransmitters, neuropeptides, short-chain fatty acids (SCFAs), and tryptophan metabolites, as well as neural pathways involving the ENS, vagus nerve, and spinal nerves [8,9,11,33].

Several mechanisms may explain how gut microbes affect the HPA axis. Gut dysbiosis may lead to the release of cytokines such as IL-1β, IL-6, and TNF-α, which can cross the blood–brain barrier (BBB) and activate the HPA axis. Bacterial components like lipopolysaccharide (LPS) and peptidoglycan can also stimulate this system. Additionally, *E. coli* can produce the ClpB protein, which mimics α-MSH and promotes the production of ACTH by stimulating the release of proopiomelanocortin [34].

Metabolites produced by the gut microbiota can significantly influence the brain–gut axis. For example, SCFAs like butyrate, produced by the microbiota, inhibit histone deacetylases, supporting memory and neural plasticity. A study by Resende et al. (2013) suggests that butyrate may have mood-stabilizing effects in rodent models, such as reducing depressive-like behavior induced by chronic psychosocial stress and reversing anhedonia and sociability impairments [35]. Similarly, propionate helps protect the BBB from oxidative stress. SCFAs also affect neuroinflammation by modulating the proliferation of immune cells, such as T-cells and neutrophils, and influencing the production of inflammatory cytokines [16,21,33]. Recent clinical evidence from a randomized, placebo-controlled trial demonstrates that colon-delivered SCFAs can alter the HPA axis responsivity to psychosocial stress [36,37].

The microbiota also secretes a variety of neurotransmitters. For example, *Lactobacillus* subspecies can produce acetylcholine., *Candida*, *Streptococcus*, *Escherichia coli*, and *Enterococcus* secrete 5-HT, while *Bacilli* and *Serratia* secrete dopamine. *Lactobacillus acidophilus*, *Bifidobacterium infantis*, *Bifidobacterium*, *Candida*, and *Streptococcus*, have demonstrated therapeutic effects on mental health by secreting neurotransmitters like gamma-aminobutyric acid (GABA), serotonin, glycine, and catecholamines or by modulating endocannabinoid expression [2,10,33].

The gut microbiota also metabolizes tryptophan, which is a precursor for indoles, serotonin, and melatonin synthesis, thereby limiting tryptophan availability for the host. *Pseudomonas* species synthesize serotonin from tryptophan, which is involved in toxicity and intercellular signaling. As a result, the reduction in circulating tryptophan due to gut microbiota affects serotonergic transmission and the functioning of both the CNS and the enteric nervous system [33]. Kynurenine, the other metabolite, is part of the kynurenine pathway (KP), which under normal conditions maintains immune function and neurotransmitter balance. Fortunately, increased gut permeability and systemic inflammation can dysregulate KP activity and result in elevated production of neurotoxic metabolites such as quinolinic acid. These metabolites affect the central nervous system (CNS) by inducing oxidative stress, possibly causing neuronal damage and the progression of neurological disorders. *Lactobacillus* species can influence kynurenine synthesis by producing hydrogen peroxide, which suppresses the expression of indoleamine 2,3-dioxygenase (IDO1)—the enzyme responsible for converting tryptophan (Trp) to kynurenine in the gastrointestinal tract. Chronic stress has been shown to reduce *Lactobacillus* abundance in rodent models, resulting in decreased hydrogen peroxide levels, upregulation of IDO1, and increased kynurenine production, which correlates with depression-like behaviors [38,39,40,41,42]. Moreover, essential vitamins, such as vitamins K, B2, B9, and B12, synthesized by the microbiota, have neuroprotective effects on the central nervous system (CNS) [33].

Moreover, essential vitamins, such as vitamins K, B2, B9, and B12, synthesized by the microbiota, have neuroprotective effects on the central nervous system (CNS) [33].

**Figure 3:**
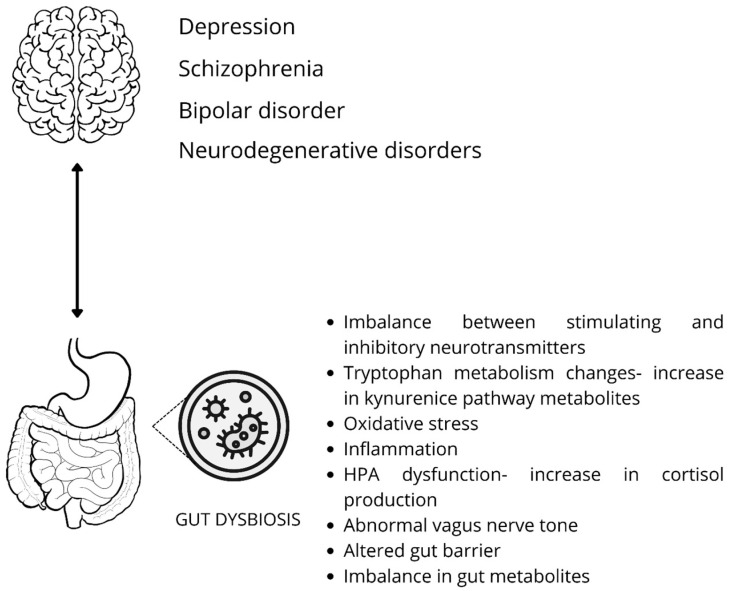
Dysbiosis of the gut microbiota can disrupt the brain–gut axis and affect mental health through several interconnected processes. These include decreased serotonin production due to impaired tryptophan metabolism and a lack of essential micronutrients; oxidative stress and neuroinflammation caused by increased reactive oxygen species (ROS) and microbial imbalance; disruption in neurotransmitter balance, including reduced inhibitory signals and altered excitatory transmission; dysfunction of the hypothalamic–pituitary–adrenal (HPA) axis, leading to excessive cortisol release and an exaggerated stress response; impairment of the intestinal barrier, allowing endotoxins to enter the bloodstream and trigger immune activation; and vagus nerve dysfunction, which weakens communication between the gut and brain. Together, these factors create a neuroinflammatory environment that may contribute to the development of psychiatric conditions such as depression and anxiety [8,9,10,11,21,26,27,33,34].

These intricate interactions highlight the gut–brain axis as a key pathway through which alterations in gastrointestinal function may contribute to the development and progression of psychiatric conditions. A schematic diagram of the brain–gut axis theory is shown in Figure 3.

## 4. Psychiatric Disorders Associated with Gut Microbiota

Gastrointestinal tract can influence not only development but also the course of diseases. In this paper, the diseases, associated with microbiota, are divided into psychiatric disorders (bipolar disorder, schizophrenia, depression), neurodegenerative diseases (Alzheimer’s disease and Parkinson’s disease) and neuroatypical conditions such as autism spectrum disorder (ASD) and attention-deficit/hyperactivity disorder (ADHD).

### 4.1. Bipolar Disorder

Bipolar Disorder (BD) is a mental health condition defined by repeated cycles of depressive and elevated mood states, often accompanied by notable changes in energy, activity levels, cognition, and behavior. It is recognized as a major contributor to global disability and is strongly associated with elevated risks of early death. Globally, approximately 15–20% of people with bipolar disorder die by suicide, with 30–60% making at least one attempt [43]. Studies suggest that individuals with BD experience notable shifts in the composition of their gut microbiota. Although alterations were observed, they did not exhibit a consistent pattern [44,45,46].

Huang et al. (2023) identified significant differences in the gut microbiota composition of patients with BD [47]. The study included a total of 72 BD patients experiencing depressive episodes and 16 healthy controls (HCs). While the overall microbial diversity did not differ significantly between the two groups, distinct variations in taxonomic composition were observed. Specifically, the relative abundance of *Bacilli*, *Lactobacillales*, and the genus *Veillonella* was elevated in BD patients compared to HCs, whereas the genus *Dorea* was found to be more prevalent in the control group. Furthermore, correlation analysis revealed that the abundance of certain bacterial generation in BD patients was significantly associated with both the severity of depressive symptoms and levels of inflammatory markers [47].

Gut microbiota alterations in bipolar disorder (BD) have been linked to disruptions in key metabolic pathways, including tryptophan (Trp) synthesis and metabolism. Using shotgun metagenomic sequencing (SMS), Lai et al. (2021) analyzed fecal samples from 25 BD patients and 28 healthy controls (HCs) to investigate differences in gut microbial composition and Trp-related gene expression [48]. Results showed altered tryptophanase and aromatic aminotransferase activity, which led to reduced tryptophan availability and, consequently, lower serotonin levels in individuals with BD. BP patients showed a significant decrease in the abundance of the phylum *Bacteroidetes*, while *Actinobacteria* and *Firmicutes* were notably increased compared to HCs [48].

In a study conducted by Coello et al. (2019), gut microbiota composition was analyzed in 113 patients with bipolar disorder (BD), 39 unaffected first-degree relatives, and 77 healthy controls (HCs) [49]. The researchers identified 64 bacterial generations, with *Flavonifractor* showing a significantly higher prevalence in BD patients (61%) compared to HCs (39%) (*p* = 6.3 × 10^−4^, Q = 0.04). Importantly, newly diagnosed BD patients had a 2.9-fold increased likelihood of *Flavonifractor* detection relative to HCs (OR = 2.9, 95% CI: 1.6–5.2, *p* = 5.8 × 10^−4^, Q = 0.04) [49].

Painold et al. (2019) identified distinct gut microbiota patterns in BP patients, particularly in relation to depressive symptoms [50]. Patients in a depressive state showed levels higher of *Enterobacteriaceae* (LDA = 3.12, *p* = 0.044), while those with symptom improvement had greater abundance of *Clostridiaceae* (LDA = 3.41, *p* = 0.048) and *Roseburia* (LDA = 3.13, *p* = 0.016). When compared to HCs, BD patients exhibited increased levels of *Actinobacteria* (LDA = 4.82, *p* = 0.007) and *Coriobacteria* (LDA = 4.75, *p* = 0.010), whereas *Ruminococcaceae* (LDA = 4.59, *p* = 0.018) and *Faecalibacterium* (LDA = 4.09, *p* = 0.039) were more prevalent in controls [50].

A difference in *Faecalibacterium* spp. was also found in one of the first studies on this matter, conducted by Evans et al. [51].

Notably, Aizawa et al. (2019) found no significant differences in fecal *Bifidobacterium* or *Lactobacillus* counts between BD patients and controls [52]. However, the study did reveal a notable negative correlation between *Bifidobacterium* levels and cortisol, suggesting a potential link between gut bacteria and HPA axis activity [52]. Changes in the qualitative composition of microbiota diversity during the development of BD are shown in Figure 3.

The potential of gut microbiota modulation as an adjunctive approach in bipolar disorder (BD) was examined in a cohort study conducted by Reininghaus et al. (2020) [53]. The study involved 20 euthymic individuals with BD who received probiotic supplementation over a 3-month period. Cognitive performance was assessed using a standardized neuropsychological battery. The findings revealed a significant improvement in attention and psychomotor processing speed, as evidenced by higher scores on the Digit Symbol Test after both one month (t2) and three months (t3) of treatment (F = 8.60; η^2^ = 0.49, *p* < 0.01). Furthermore, executive functioning, measured by the Trail Making Test Part B (TMT-B), also improved significantly over the course of the intervention (F = 3.68; η^2^ = 0.29, *p* < 0.05) [53].

The use of probiotic supplementation was also investigated by Eslami Shahrbabaki et al. (2020) in a randomized, placebo-controlled trial involving patients with type 1 bipolar disorder [54]. 38 participants were allocated to a probiotic or placebo group using blind randomizing method [54]. Mood symptoms were assessed at baseline, week 4, and week 8 using the Young Mania Rating Scale (YMRS) and HAM-D. While both scales showed greater reductions over time in the probiotic group compared to placebo, the differences were not statistically significant [54].

**Figure 4:**
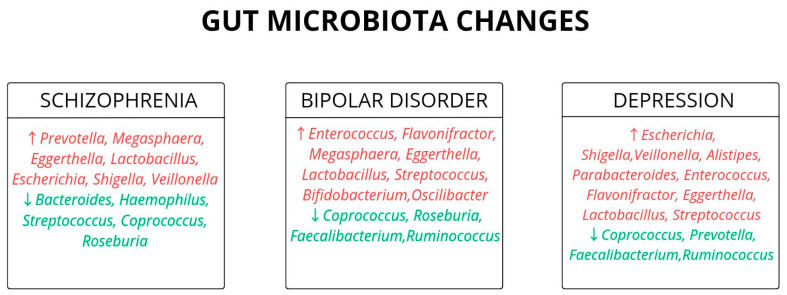
Described changes in gut microbiota in Bipolar Disorder, Schizophrenia and Major Depressive Disorder. These imbalances are associated with disrupted metabolism of neurotransmitters like GABA and glutamate, overproduction of lactic acid, mitochondrial dysfunction, and systemic acidosis. Such changes may contribute to neuroinflammation, altered brain pH, and increased risk of mental disorders [15,44,45,46,49,55,56,57,58,59,60,61,62].

Changes in the qualitative composition of microbiota diversity during the development of BD are shown in Figure 4. A summary of the most relevant information contained in the reviewed literature of BD is presented in Table 1.

### 4.2. Schizophrenia

Schizophrenia (SCZ) is a complex, multifactorial neurodevelopmental disorder that typically manifests in adolescence, affecting approximately 0.20 per 1000 people annually. It is characterized by symptoms such as hallucinations, delusions, disorganized speech and behavior, and cognitive decline. While the exact causes remain unclear, emerging evidence suggests a potential link between gut microbiota alterations and SCZ, particularly given the high prevalence of gastrointestinal issues in affected individuals [15,21,64,65].

Several studies have reported a decreased abundance of *Firmicutes* and inconsistent findings regarding *Proteobacteria* [55,66]. In drug-naïve first-episode psychosis patients, reduced levels of *Bifidobacterium* spp., *E. coli*, and *Lactobacillus* spp. were observed [15], although some studies found increased *Lactobacillus* levels, which correlated with more severe positive symptoms and worse functioning [67].

Zhu et al. (2020) identified elevated levels of anaerobic and oral cavity-associated bacteria in the intestines of SCZ patients, with transplantation of *Streptococcus vestibularis* into mice inducing SCZ-like behaviors [56]. Additionally, a study by Zheng et al. (2019) found that fecal microbiota transplantation (FMT) from patients with SCZ also induced SCZ-like behaviors in mice [57]. The study identified a microbial panel—compressing *Aerococcaceae*, *Bifidobacteriaceae*, *Brucellaceae*, *Pasteurellaceae*, and *Rikenellaceae*—which demonstrated strong discriminatory power in differentiating SCZ patients from healthy controls. GF mice receiving SCZ microbiome transplants exhibited lower hippocampal glutamate alongside elevated glutamine and GABA levels, neurotransmitter alterations consistent with glutamatergic hypofunction implicated in SCZ pathophysiology. Furthermore, the abundance of *Veillonellaceae* and *Lachnospiraceae* taxa was significantly correlated with the severity of symptoms, as measured by the Positive and Negative Syndrome Scale (PANSS) [56,57].

The role of specific bacterial strains in influencing schizophrenia-related traits is not yet well defined. Probiotics are being investigated as a potential therapeutic option. In a randomized controlled trial by A. Ghaderi et al., patients with schizophrenia received a probiotic supplement containing *Lactobacilli* and *Bifidobacterium bifidum* together with vitamin D. The intervention reduced C-reactive protein (CRP) levels, increased plasma total antioxidant capacity, and improved general and total PANSS scores, indicating reduced inflammation. Nonetheless, it was unclear which component was responsible for the improvement [68]. Changes in the qualitative composition of microbiota diversity during the development of schizophrenia are shown in Figure 4. A summary of the most relevant information contained in the reviewed literature of SCZ is presented in Table 2.

### 4.3. Depression—A Global Disease

An increasing number of studies are focusing on the impact of diet on the risk of developing depression and anxiety disorders. This is important for expanding our understanding of the factors contributing to the development of these disorders, as they are considered among the top 10 leading causes of global disease burden [72,73].

The Anxiety and Depression Association of America reported that depression is the most common mental disorder worldwide—affecting nearly 264 million people [73].

Compared to 2017, the global prevalence of depression has increased approximately sevenfold, a rise attributed in part to the COVID-19 pandemic [73,74].

Depression and anxiety disorders have a negative impact not only on the quality of life of patients but also on healthcare systems, creating significant social and economic issues. Therefore, prevention and treatment of these conditions should be a global health priority [58,72,75].

#### 4.3.1. Microbiome–Gut–Brain Axis (MGBA) in Depression

The exact mechanisms of the gut–brain axis are not yet fully understood, although research highlights the crucial role of hormonal, metabolic, immunological, and neural signals—involving the central, autonomic, and enteric nervous systems [8,58,72].

The gut–brain relationship is considered bidirectional. On one hand, the gut microbiota (closely linked to diet) influences the brain by modulating gene expression, affecting neurotransmitter pathways (serotonin, dopamine, glutamate, and GABA), regulating neuroinflammation, and producing IGF-1. Additionally, the microbiota affects the BBB, and dysbiosis increases its permeability [8,58]. On the other hand, stress can negatively affect dietary habits, temporarily altering the gut microbiota composition and modifying gut function and permeability [8,58,59,72].

The close connection between gut function and mental health is further confirmed by the effectiveness of antidepressants in treating irritable bowel syndrome (IBS), and by the fact that gastrointestinal disturbances are among the most common physical symptoms associated with depression [8,76].

It has been shown that individuals with mental disorders exhibit altered gut microbiota composition compared to healthy individuals [58,64,72,76,77].

In patients with depression, an increased abundance of bacteria such as *Alistipes*, *Escherichia*, *Lactobacillus*, *Veillonella*, *Enterococcus*, *Flavonifractor*, *Eggerthella*, *Streptococcus*, and *Parabacteroides* has been observed, along with a reduced presence of *Prevotella*, *Coprococcus*, *Faecalibacterium*, and *Ruminococcus* [76]. These changes in gut microbiota may contribute to the development of mental disorders, likely through increased production of lactic acid, butyrate, and interference with glutamate and GABA metabolism [58,76].

The development of various psychiatric disorders has also been linked to impaired energy production in mitochondria, leading to elevated lactate levels and increased systemic acidity. Gut bacteria in patients with psychiatric disorders may also contribute to brain pH reduction. Some of these bacteria produce lactic acid (e.g., *Lactobacillus*, *Enterococcus*, *Streptococcus*), while others, in a compensatory mechanism, use lactic acid for their metabolism (e.g., *Escherichia*, *Veillonella*) [76].

Although these bacteria typically benefit the host by regulating metabolism, protecting against pathogens, and modulating immune responses [60,61,76], lactate accumulation in the gut may have negative effects, potentially leading to acidosis, cardiac arrhythmias, and neurotoxicity [62,76].

Furthermore, the digestive system is the largest immune organ in the body. Therefore, disruptions in gut microbiota composition may impair Treg lymphocyte function and contribute not only to the development of psychiatric disorders but also to autoimmune, inflammatory, and allergic diseases [8]. Changes in the qualitative composition of microbiota diversity during the development of Major Depressive Disorder are shown in Figure 4.

#### 4.3.2. Dietary Patterns Linked to Depression

Many existing studies indicate a lower likelihood of depression in individuals who follow current healthy eating guidelines, such as adhering to a Mediterranean diet [58,72,75,78].

This diet is based on fruits, vegetables, grains, nuts, and olive oil. These foods are characterized by a high antioxidant and anti-inflammatory potential. They promote diversity in gut microbiota and proper gut barrier function [78].

It has been shown that this type of diet reduces the level of pathogenic bacteria, such as *Proteobacteria*, while increasing beneficial bacteria (e.g., *Bifidobacteria*, *Clostridium* cluster XVIa) [72]. An important component of the diet is dietary fiber, which, by stimulating the production of SCFAs, promotes the development of “beneficial” bacteria [58,75].

**Figure 5:**
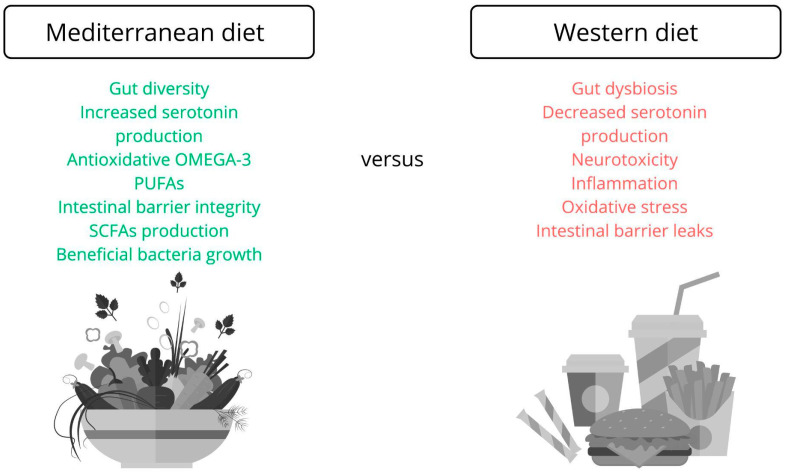
Differences due to dietary habits in the functioning of the microbiome and the gut–brain axis. The Mediterranean diet—rich in fruits, vegetables, whole grains, nuts, and olive oil—supports mental well-being through its antioxidant and anti-inflammatory properties. It boosts serotonin production, maintains gut barrier integrity, promotes beneficial bacteria, and increases short-chain fatty acid (SCFA) production. It also helps reduce harmful bacteria like *Proteobacteria*. In contrast, the Western diet—high in processed foods, sugars, and saturated fats, and low in fiber—is linked to gut dysbiosis, inflammation, and a higher risk of mood disorders. This comparison highlights how diet directly impacts gut health and brain function, playing a key role in mental well-being [58,72,75,78].

In contrast, the Western diet, based on highly processed foods with a high content of simple sugars and saturated fats, and low fiber, is associated with increased inflammation, gut dysbiosis, and neurotoxicity. Studies show that individuals following a Western diet have a higher risk of developing mood disorders than those adhering to health-promoting dietary models [75,78]. This is summarized in Figure 5.

#### 4.3.3. Potential Pathogenesis of Depression: The Connection to Diet and Microbiome

Although the exact mechanisms leading to depression are not fully known, we are aware of certain neurobiological changes strongly linked to the disease.

The most well-known change is the reduction in serotonin levels in the brain.

Peripheral serotonin, although it does not cross the blood–brain barrier, influences intestinal peristalsis, the immune system, and the function of the enteric nervous system [72,78]. Approximately 90% of this neurotransmitter is produced in the gastrointestinal tract [78,79].

Its precursor is tryptophan, and the conversion of tryptophan to serotonin is promoted by a balanced microbiome. Disruptions in tryptophan metabolism lead to neurotoxic products from the kynurenine pathway, which are associated with depression [58,80]. It has been shown that micronutrients present in the diet, such as zinc, magnesium, iron, vitamin B12, and folic acid, influence serotonin levels [72]. Gut microbiota can influence the balance between excitatory and inhibitory neurotransmitters through the production of their precursors, modulation of enzymes involved in their synthesis, and interaction via the gut–brain axis. Consequently, gut dysbiosis contributes to mood fluctuations, stress responses, and the development of psychiatric disorders such as depression and anxiety [8,58,72,78,81].

A major factor in the development of mood disorders—particularly depression and anxiety—is oxidative stress. This condition is associated with elevated levels of reactive oxygen species (ROS), which may lead to neuronal damage, heightened inflammation, disrupted neurotransmission, and increased intestinal permeability Dysbiosis, commonly observed in depressive disorders, significantly contributes to oxidative stress both locally in the gut and systemically [5,8,58,72].

Another important mechanism involved in depression is the dysfunction of HPA axis, which plays a key role in the body’s stress response, primarily through the release of cortisol [58,72,78]. The gut microbiota has a significant impact on HPA axis activity. Through the production of SCFAs and other bioactive compounds, gut microorganisms reduce inflammation and help prevent overstimulation of the HPA axis [77,78]. Microbial imbalance in the gut is associated with the translocation of endotoxins into the bloodstream, triggering immune responses and activation of the HPA axis. This leads to cortisol overproduction, which has been linked to depression and neurodegenerative processes [78,82].

Diet also modulates these mechanisms. A diet rich in fruits and vegetables contains beneficial micronutrients and antioxidants (e.g., vitamin C, polyphenols, flavonoids), which have been shown to exert antidepressant-like effects [72,75]. Omega-3 polyunsaturated fatty acids (PUFAs), found in plant-based foods, support the nervous system by improving membrane fluidity and permeability [59]. They also reduce brain inflammation and lower cortisol levels, enhancing the body’s resilience to stress [78,83]. On the other hand, high intake of refined sugars and saturated fats has been linked to elevated cortisol levels and dysregulation of the HPA axis [84]. Deficiencies in micronutrients such as magnesium and vitamin B6 may also impair the stress response and increase cortisol levels [78,82]. Considering the above, a healthy, balanced diet may lower the risk of developing depression [59,72,78]

Bacterial metabolites produced by gut microbiota also contribute to the development of anxiety and depressive disorders. Some of these metabolites—such as gamma-aminobutyric acid (GABA), acetylcholine, serotonin, and norepinephrine—directly affect synaptic transmission and play a crucial role in mood and behavior regulation [58,78,81]. Additionally, gut bacteria produce SCFAs such as butyrate, which support intestinal barrier integrity, reduce systemic inflammation, and promote proper serotonergic signaling in the central nervous system [58,85]

Another potential contributor to depression is vagal nerve dysfunction, which disrupts communication between the gut and the brain [8].

#### 4.3.4. Fecal Microbiota Transplantation (FMT) and Depressive Disorders

Patients with neurological and neurodegenerative diseases exhibit changes in the composition of gut bacteria [8,67]. The toxins and other metabolites produced by these bacteria interact directly with the intestinal epithelium, increasing gut barrier permeability. This leads to chronic inflammation and activation of immune cells in the gut [86].

Fecal microbiota transplantation (FMT) involves administering gut flora from healthy donors to patients, resulting in a rapid transformation of the recipient’s dysbiotic microbiota [78,86,87]. This method is gaining increasing popularity, particularly the capsule form, in which patients ingest a carefully prepared fecal preparation orally—offering a less invasive alternative to endoscopic delivery [88]. Most studies involving FMT have shown a reduction in depressive-like behaviors. The transplanted healthy microbiota suppresses neuroinflammation, balances gut bacteria, and restores intestinal barrier function [78,87]. Its effectiveness has been demonstrated in patients suffering from both inflammatory bowel disease (IBD) and depression. Even when gastrointestinal symptoms did not fully resolve, FMT therapy progressively alleviated depressive symptoms in these patients [78,88,89]. A summary of the most relevant information contained in the reviewed literature of depression is presented in Table 3.

## 5. Neurodegenerative Disorders

The rising incidence of neurodegenerative diseases poses a growing global health challenge. Although the underlying mechanisms are not yet fully understood, increased intestinal permeability and disturbances in the kynurenine pathway are thought to significantly contribute to disease development. This opens new therapeutic possibilities—not only focusing on symptom management, but more importantly, on addressing root causes [90].

### 5.1. Dementia

Dementia is characterized by memory impairment and cognitive dysfunction, disrupting patients’ daily functioning. In 2021, approximately 57 million people worldwide were living with dementia, with around 10 million new cases diagnosed each year [91,92]. Recent studies suggest a link between gut microbiota and dementia [8,91,93].

### 5.2. Alzheimer’s Disease

Alzheimer’s disease (AD) is the most common cause of dementia, accounting for 60–70% of cases [91]. It is a progressive neurodegenerative disorder marked by memory loss and cognitive decline. Pathologically, it is characterized by the presence of amyloid plaques formed from misfolded beta-amyloid (Aβ) protein and neurofibrillary tangles composed of hyperphosphorylated tau protein [91,94]. Genetic mutations, combined with environmental factors, are believed to contribute to AD development [91]. Recent research indicates that gut dysbiosis may trigger beta-amyloid accumulation in the brain, contributing to the onset of Alzheimer’s disease [91,94,95]. Proposed mechanisms include increased production of pro-inflammatory cytokines, decreased levels of neuroprotective SCFAs, and heightened blood–brain barrier permeability [91,94,95]. The translocation of bacterial lipopolysaccharides into the bloodstream and subsequently into the brain induces neuroinflammation and insulin resistance, further increasing AD risk [96]. Neurotoxic metabolites from the kynurenine pathway also play a significant role in AD by promoting abnormal amyloid plaque formation [90]. The co-occurrence of inflammation caused by gut microbiota imbalances, aging processes, and poor nutrition in elderly individuals may significantly contribute to Alzheimer’s development. Thus, modulating the gut microbiota through a targeted diet or probiotic supplementation may offer new preventive and therapeutic strategies for this disease [95].

### 5.3. Parkinson’s Disease

Parkinson’s disease (PD) is the second most common neurodegenerative disorder worldwide, after Alzheimer’s disease (AD) [91].

Its characteristic symptom is motor dysfunction resulting from a reduced level of dopamine [91,97]. An increasing amount of scientific evidence points to a relationship between dysbiosis and the risk of developing PD, supported by the observation that Parkinson’s disease is often preceded by gastrointestinal symptoms [91,98]. Studies show differences in the gut microbiota composition in Parkinson’s patients compared to healthy individuals [99]. Among the findings, there is a reduction in the number of *Faecalibacterium* species that produce SCFAs, along with an increased presence of bacteria from the Lactobacillus, Akkermansia, and *Bifidobacterium* genera [92,99]. This dysbiosis could contribute to increased gut permeability, inflammation, and consequently the accumulation of α-synuclein protein (Lewy bodies), leading to the death of dopamine-producing neurons [91,99]. The kynurenine pathway also plays a significant role in the pathogenesis of PD, as dopaminergic neurons in the substantia nigra are particularly vulnerable to toxic metabolites of this pathway. Therefore, the development of therapeutic strategies aimed at modulating the kynurenine pathway is needed to limit neurodegeneration and shift the metabolic balance toward neuroprotective compounds such as kynurenic acid [90].

Despite these observations, the definitive link between the microbiome and Parkinson’s disease has not yet been fully confirmed, and further research in this area is needed [90,91].

### 5.4. FMT (Fecal Microbiota Transplantation) and Neurodegenerative Diseases

Studies in mice with Parkinson’s disease have shown that FMT (fecal microbiota transplantation) reduces dysbiosis, alleviates motor impairment, and increases dopamine and serotonin levels in the striatum of the mice by suppressing neuroinflammation and reducing TLR4/TNF-α signaling [86,98]. Gut dysbiosis also contributes to the pathogenesis of Alzheimer’s disease. Some populations of gut microbes promote amyloid formation, which could lead to the development of Alzheimer’s. By altering the composition of the gut microbiota, there is hope for slowing the progression of Alzheimer’s disease [86,95]. Further research in this area involving human participants is necessary, but the results of animal studies so far raise hope for a new therapeutic approach for neurodegenerative diseases [86,98].

## 6. Neuroatypical Conditions

Neuroatypical an neurodivergence disorders (NACs) are conditions in which cognitive abilities, learning-related skills, emotions, attention, social skills, and other mental functions are developed differently than those in the general population. Patients with NACs as they age, experience further cognitive decline due to multiple pre-existing cognitive, thought, and sensory impairing conditions. Conditions include a range of disorders affecting intellectual development, mental health, communication, and motor or brain function [38,39].

Neurodivergence, which is a more narrow term than neuroatypical, refers to variations in mental or neurological function from what is considered typical and acknowledges that there are many ways in which people experience life and interact with others. It was traditionally diagnosed more in males but is now increasingly recognized in females, often diagnosed later due to their tendency to mask symptoms to avoid social exclusion. Among this term we incorporate ASD, ADHD and Tourette’s syndrome [39].

Autism and ADHD are neurodevelopmental disorders that often co-occur, impacting emotional, social, and psychological activity [40]. Distinct cortical patterns, modulated by age, sex, and the potential co-occurrence of these two neuroatypical disorders, were also observed [41].

### 6.1. Autism Spectrum Disorder (ASD)

Autism spectrum disorder (ASD) refers to a group of neurodevelopmental disorders characterized by difficulties in communication and the development of social relationships. The prevalence of ASD is continuously increasing [8,33,42,100,101]. Over 70% of patients with this disorder report gastrointestinal symptoms, which is why ASD is increasingly considered in the context of the gut–brain axis [100,102,103,104]. Changes in gut microbiota composition may contribute to the activation of the immune system and dysregulation of this axis in individuals with ASD [101,103,105,106]. According to some authors, gastrointestinal symptoms may correlate with the severity of autism symptoms [8,107]. This could be related to the frequent occurrence of “leaky gut” in ASD patients, which allows bacterial toxins to pass into the bloodstream more easily, negatively affecting brain function and, consequently, social behaviors [91,108].

In addition to gastrointestinal complaints, ASD patients often have altered gut microbiota composition [8,42,91,109,110]. Studies conducted on children with ASD have identified differences in gut microbiota composition compared to healthy children [33,64,91,104].

There was a noted reduction in microbiota diversity, lower levels of Bifidobacterium, *Bacteroides*, *Ruminococcus*, *Prevotella*, *Coprococcus*, and *Veillonellaceae*, and higher levels of *Lactobacillus*, *Desulfovibrio*, *Alistipes*, *Akkermansia*, and *Sutterella* bacteria [7,91,100,109,111,112]. Higher levels of Clostridium histolyticum, known for producing neurotoxins, have also been reported [91,113]. These bacteria also produce propionic acid (a SCFA), which is likely associated with ASD [91]. Sutterella bacteria degrade immunoglobulin A in mucosal membranes, contributing to epithelial cell damage in the gut. They also induce inflammation through the production of interleukin-8 [114]. Lower levels of Bacteroides also promote the development of inflammation in the gut. These findings confirm disrupted gut microbiota composition and function in ASD [91,114]. Recent studies show that gut dysbiosis is associated with microglial activation in various brain regions, influencing the induction of neuroinflammation and dysfunction of neural networks [106,114]. Dysbiosis may also lead to neurochemical abnormalities, such as changes in levels of GABA, glutamate, serotonin, and oxytocin, which are also linked to the etiology of ASD. Numerous studies indicate that deficiencies in some of these neuromodulators, such as serotonin and oxytocin, correlate with social problems characteristic of ASD [102,114].

It has been observed that children with ASD have significantly lower levels of KA and higher QA levels in serum compared to typically developing children. The KA and QA are products of kynurenine metabolism described in Section 4.3.3. This suggests an increased potential for neurotoxicity, which may have significance in the pathophysiology of this disorder [103]. Studies on patients with ASD show that probiotics containing strains from *Lactobacillus* and *Bifidobacterium* help alleviate not only gastrointestinal issues but also behavioral abnormalities in individuals with ASD. However, the current state of knowledge regarding the efficacy and safety of these products requires further confirmation [42,101,102,104,105].

An important aspect of alleviating ASD symptoms is diet. It is recommended to limit the intake of hard-to-digest proteins, which can lead to the production of harmful by-products, and to avoid a high-fat diet. Vitamin D supplementation may also be beneficial for these patients [103,106].

In studies conducted on children with autism, temporary improvements were observed in communication, stereotypy, anxiety, and sensory-motor behaviors after microbiota therapy [105,107].

Reconfiguring gut microbiota is an innovative, promising strategy for alleviating inflammation and gastrointestinal complaints, which are commonly associated with ASD [100,101,104,105].

### 6.2. Attention-Deficit/Hyperactivity Disorder (ADHD)

ADHD is a neuropsychiatric disorder characterized by inattention, impulsivity, and hyperactivity [8,33]. It affects approximately 6 million children aged 3 to 17 years [58]. The causes of ADHD are attributed to impaired functioning of neurotransmitters such as dopamine and norepinephrine [33,58,115]. It is suggested that changes in gut microbiota may be linked to the development or exacerbation of ADHD symptoms [8]. This connection arises from the production of dopamine and norepinephrine precursors by certain gut bacteria, which contribute to the development of this disorder [33].

Studies conducted on patients with ADHD have observed a higher number of *Bifidobacterium* bacteria in the gut compared to control groups. Increased activity of cyclohexadienyl dehydrase, which is associated with the synthesis of dopamine precursor phenylalanine, was also noted [33,58,116].

It can be assumed that specific dietary components, through their impact on gut microbiota, influence brain areas responsible for cognitive processes and behaviors, suggesting the potential for alleviating ADHD symptoms through a special diet [117,118].

Healthy eating is of significant importance—removing artificial food colorants from the diet and increasing omega-3 fatty acid intake contributes to reducing impulsivity and improving concentration. Growing evidence also indicates that early supplementation with probiotics can reduce the risk of developing ADHD [58,119].

In neurodivergent adults, joint hypermobility, dysautonomia, and pain are commonly linked. Recognizing these connections can improve symptom management and future care strategies [40].

## 7. Role of Probiotics, Prebiotics, and Synbiotics in the Therapy of Psychiatric and Neurodevelopmental Disorders

Probiotics are live microorganisms that, when administered in adequate amounts, provide health benefits to the host. Prebiotics are nutrients that gut bacteria use to support the health of the host. Synbiotics are a combination of probiotics and prebiotics. It is believed that simultaneous administration of probiotics and prebiotics increases the activity of beneficial gut bacteria, provided that their composition and quantity are appropriately selected [78,87].

The term “psychobiotic” encompasses all three of the above terms, which influence neurotransmitters in the gut [73]. Recently, much discussion has centered around their potential use in the treatment of psychiatric and neurodevelopmental disorders such as depression, anxiety, autism, schizophrenia, and bipolar disorder [58,73,87]. This has sparked both interest and controversy. These products are promoted as substances that naturally improve mood by modulating gut microbiota. The action of psychobiotics includes regulating the immune system, producing SCFAs, and supporting the integrity of the gut barrier. They are also easily accessible—no prescription is needed to purchase them, and their sale is not subject to strict legal regulations [73].

Although the studies included in reviews suggest that probiotics can be an effective adjunct in treating psychiatric disorders, the efficacy and risks of their long-term use remain unknown. It is important that future research on psychobiotics, in addition to the role of gut microbiota, take into account factors such as geographic region, age, and comorbidities in patients. Most of the available studies suffer from low statistical power and poor methodology. Therefore, it is necessary to conduct further well-designed, randomized studies to strengthen existing evidence and create optimal protocols for the use of prebiotics/probiotics or synbiotics in the treatment of psychiatric disorders [73,78,87,103,104,105,106].

### Personalized Nutrition

The role of the gut microbiome in controlling emotions that shape our behaviors is increasingly emphasized in contemporary research. Since diet and gut microbiome composition are closely linked, there is hope for the development of specialized diets that could prevent the development of affective disorders, such as depression [120,121]. Magzal et al. prove that elderly patients can benefit from personalized dietary recomendattions [121]. Among those patients dietary interventions reduced depression symptoms, improved quality of life and increased the diversity in the gut microbiom. Two independent meta- analyses reported that elimination diets have been effective among children with ADHD and concluded that one third of them were responsive (at least 40% reduction in symptoms) [122]. Attention is paid not only to full nutrition plans, but also to individual substances in the diet. The effect of folic acid and its supplementation on depression, omega-3 fatty acids in cognitive disorders, probiotics in anxiety disorders or the effect of glycemic levels on bipolar disorder are being studied [121,122,123,124]. This is an example of modern nutritional therapy, which would take individual differences into account, forming the basis for predicting anthropometric traits based on the microbiome. This futuristic solution would require personalized examination of a patient’s gut microbiota, metabolic capacities, and genetic predispositions.

A meta-analysis of 16 eligible randomized controlled trials with outcome data for 45,826 participants concluded that dietary changes may be a promising and innovative approach to reducing depressive symptoms in the general population. Diet can play a significant role in the treatment and self-management of symptoms, and women, in particular, have demonstrated greater benefits from dietary changes in conditions such as depression and anxiety. Therefore, further research should be conducted to clarify the impact of diet on mental health [125].

Another study, which lasted 12 weeks and included 166 participants, showed that changing the diet can be a widely available option for all patients and, at the same time, be highly effective [126]. A 12-week, open-label, randomized study of men aged 18 to 25 examined the effects of a Mediterranean diet on reducing depressive symptoms. A mean reduction of 20.6 points on the depression scale was observed for the group following the Mediterranean diet. Furthermore, 36% of participants in this group reported low or minimal depressive symptoms at the end of the study. Therefore, it can be concluded that the Mediterranean diet may be effective in young men with moderate to severe clinical depression [127].

Such an approach to treatment via nutrition could provide an attractive alternative or an adjunct to current treatments for patients with mood disorders [7,8,72,75,76,78,104].

## 8. Limitations, Challenges and Future Directions

This review is not without limitations. The available data on the subject is limited, and due to the early stages of the field, the findings are often inconsistent. Many studies have small sample sizes or depend on animal models. Additionally, differences in diagnostic criteria, microbiota sequencing platforms, and data analysis methods across studies make it difficult to draw definitive conclusions. There is a clear need for standardized biomarkers in this area of study to ensure consistency, comparability, and reliability of research.

Another setback is the fact that factors like diet, environment, or genetics often cannot be fully controlled. Further research is necessary to determine how changes in the microbiome impact the development of psychiatric disorders, particularly regarding immunology or metagenomics. Additionally, it is important to consider the role of medication; however, its correlation with microbiota has been analyzed in limited studies thus far [128]. A significant number of studies do not take into account the impact of current patients’ medications. Psychiatric pharmaceuticals, such as antipsychotics, antidepressants, analgesics, and anticonvulsants, can affect gut microbiota, typically by reducing the diversity of microorganisms [129]. Y. Tomizawa et al. observed significant differences in both alpha and beta diversity among patients taking antipsychotics compared to those not on these medications, between baseline and endpoint time points [128]. Additionally, Dilomre et al. demonstrated that certain medications can be linked to specific microbes [130]. This alteration makes it hard to adequately assess the true influence of psychiatric disorders on gut microbiota and complicates the evaluation of potential gut microbiota interventions.

Moreover, while preliminary results of gut microbiome interventions are promising, their efficacy and safety need further investigation through well-designed, large-scale clinical trials. FMT presents unique challenges and is still not recognized as an acceptable therapy option by the medical community [131]. Although it is considered safe by most physicians, it can be associated with the risk of exposing the patient to enteric pathogenic microorganisms and, as a result, disease development, even if contracted from a healthy donor [132]. Two patients were reported to develop bacteremia due to ESBL-producing *E. coli* after receiving stool from the same donor for FMT. This highlights the importance of reducing the transmission of potentially harmful microorganisms. Furthermore, the stool material itself contains chemicals, metabolites, and waste, which can be potentially harmful [132].

It is also crucial to ensure treatment efficacy and stability. In various diseases, the use of microbiota intervention often results in only temporary outcomes. FMT frequently leads to a significant loss of the original microbiota. Since each individual possesses a unique and stable microbiome, it is essential to identify a healthy donor’s microbiome and ensure the reproducibility of a replica for long-term, sustainable use and stable clinical outcomes [132]. Reports suggest that multiple treatments may be necessary for a response in chronic conditions, and this response may decrease without ongoing maintenance therapy [133]. FMT regulations are yet to be standardized, which is a challenge due to difficulty in classifying and controlling the human microbiota, and a lack of adequate clinical studies. The dosage parameters of FMT, including frequency and route of administration, formulation of the material, and its processing, may depend on the condition and determine treatment success [131,133].

The procedure also raises some ethical concerns, such as obtaining informed consent from vulnerable patients, maintaining privacy, ensuring adequate protection, and selecting donors. Obtaining informed consent can be challenging due to patient vulnerabilities, the experimental nature of the method, and limited information about potential side effects [131,134]. Choosing a donor can also be difficult, as the exclusion criteria are not yet clearly defined. Additionally, during a questionnaire study, clinicians expressed significant concern about the dignity and psychological impact of FMT. However, this concern was much lower among those who had high familiarity with the procedure compared to those who did not [134,135].

While microbiota interventions show promise as a therapeutic option, further research through well-designed, large-scale clinical trials is necessary to ensure their safety, efficacy, and consistent outcomes.

There is ample evidence underscoring the crucial role of the gut microbiota in the pathophysiology and treatment of psychiatric and neurological disorders. To further deepen our understanding and explore new therapeutic solutions, future studies should be based on larger groups of individuals, as previous studies have included small numbers of subjects, significantly limiting causal inferences. Furthermore, it is worth emphasizing the importance of integrative multi-omics analyses, such as metagenomics, metabolomics, transcriptomics, and proteomics, which will be essential for unraveling complex host-microbiome interactions and identifying specific microbial metabolites, signaling pathways, and molecular mechanisms that influence brain function and behavior. Inter-individual variability in microbiota composition and tailored therapy based on this may also prove an interesting avenue. Identifying biomarkers for diagnosis would also significantly facilitate prognosis and treatment monitoring.

## 9. Conclusions

Contemporary research increasingly focuses on the correlation between diet and mental health. The findings of these studies indicate that mental disorders are not just diseases of the central nervous system (CNS). The problem also lies in many highly innervated neural networks, particularly those within the enteric nervous system. The mechanisms of the microbiome–gut–brain axis (MGBA) are complex, dependent on numerous variables, and likely based on a bidirectional relationship. We can hypothesize that genetics may influence the functioning of the microbiome–gut–brain axis.

A healthy diet impacts the composition of gut microbiota, promoting the growth of commensal bacteria and inhibiting the growth of pathogenic bacteria. Dietary interventions also influence gut wall permeability and reduce inflammation. Therefore, since microbiome composition is largely influenced by what we eat, there is a high likelihood that an improper diet affects neurobiological and emotional processes, manifesting as psychiatric disorders.

Further research is needed to specifically determine how and which changes in the microbiome influence the development of psychiatric disorders, especially considering the role of the immune system, metabolome analysis, and metagenomics.

There are increasing proposals for new forms of psychiatric disorder treatment that focus on restoring a healthy gut microbiota. These future therapeutic options could use appropriately selected probiotics, fecal microbiota transplantation, or personalized nutrition.

In addition to direct treatment of microbiota imbalances, preventive measures should also be emphasized. The composition of the gut microbiota at birth is influenced by many factors, such as prematurity, maternal age, childhood social stress, breastfeeding, parental smoking, and the presence of pets. Parents should be aware of these factors and take appropriate steps to manage them from an early age.

## Figures and Tables

**Table 1:** The major findings regarding bipolar disorder and gut microbiota reported in this article ↑ represents positive association, ↓ represents negative association Abbreviations: BD, bipolar disorder patients; HC, healthy controls; Trp, tryptophan; IL-6, interleukin 6.

Publication	Type of Study	Taxonomic Differences	Additional Information
Evans et al. (2017) [51]	Case-control study	↓ *Faecalibacterium* in BD	*Faecalibacterium* associated with improved physical health, depression, and sleep quality scores
Aizawa et al. (2019) [52]	Case-control study	No significant difference	Negative correlation between *Lactobacillus* counts and sleep. Negative correlation between *Bifidobacterium* counts and cortisol levels
Reininghaus et al. (2020) [53]	Cohort study	-	*Lactobacillus*, *Bifidobacterium*, and *Lactococcus* supplementation improved cognitive function in patients with BD
Huang et al. (2023) [47]	Cross-sectional study	↑ *Bacilli*, *Lactobacillales and Veillonella* in BD, ↑ *Dorea* in HC	Bacterial genera’ abundance in BD patients was strongly correlated with the severity of depression and inflammatory markers
Lai et al. (2021) [48]	Cross-sectional study	↓ *Bacteroidetes*, *↑ Actinobacteria* and *Firmicutes* in BD	Decreased plasma Trp levels in BD
Coello et al. (2019) [49]	Cross-sectional study	↑ *Flavinofactor* BD	-
Painold et al. (2019) [50]	Cross-sectional study	↑ *Actinobacteria* and *Coriobacteria*, ↓ *Ruminococcaceae* and *Faecalibacterium* in BD	A correlation between BD higher IL−6 levels and greater abundance of *Lactobacillus*, *Streptococcaceae*, and *Bacilli*
McIntyre et al. (2021) [63]	Cross-sectional study	↑ *Clostridiaceae* and *Collinsella* in BD	-
Shahrbabaki et al. (2020) [54]	Randomized Controlled Trial	-	No significant difference between placebo and probiotic groups

**Table 2:** The major findings regarding schizophrenia and gut microbiota reported in this article ↑ represents positive association, ↓ represents negative association Abbreviations: SP, schizophrenia patients; HC, healthy controls; FMT, fecal microbiota transplantation; GF, germ free; GABA, gamma-aminobutyric acid; FEP, first-episode psychosis; PANSS, Positive and Negative Syndrome Scale.

Publication	Type of Study	Taxonomic Differences	Additional Information
Zhu et al. (2020) [56]	Metagenome-wide association study	↑ *Veillonella atypica*, *Veillonella dispar*, *Bifidobacterium dentium*, *Dialister invisus*, *Lactobacillus oris*, *Streptococcus salivarius*, *Lactobacillus fermentum*, *Enterococcus faecium*, *Alkaliphilus oremlandii*, *and Cronobacter sakazakii/turicensis*	FMT of a schizophrenia-enriched bacterium, *Streptococcus vestibularis*, induced deficits in social behaviors and altered neurotransmitter levels in mice.
Zheng et al. (2019) [57]	Translational	↑ *Acidaminococcus*, *Akkermansia*, *Alistipes*, *Citrobacter*, *Dialister*, *Veillonella*	FMT from SP induced schizophrenia-like behaviors in GF mice and displayed lower glutamate and higher glutamine and GABA in the hippocampus
Manchia et al. (2021) [69]	Cross-sectional study	↓ *Acetanaerobacterium*, *Haemophilus*, and *Turicibacter*	A higher relative abundance of the genera *Actinomyces* and *Porphyromonas* was observed in the gut microbiota of treatment-resistant schizophrenia patients compared with those responsive to antipsychotics.
Nguyen et al. (2021) [55]	Cross-sectional study	↑ *Lachnospiraceae*	Functional pathways involving trimethylamine-N-oxide (TMAO) reductase and Kdo2-lipid A biosynthesis were altered, showing associations with inflammatory cytokines and increased coronary heart disease risk.
Castro-Nallar et al. (2015) [70]	Cross-sectional study	↑*Firmicutes Ascomycota*, *Bifidobacterium* and *Lactobacilli ↓Bacteroidetes* and *Actinobacteria*	In SP, the oral microbiome showed reduced biodiversity and a higher prevalence of metabolic pathways related to metabolite transport systems, such as those for siderophores, glutamate, and vitamin B12, whereas HC exhibited greater abundance of pathways involved in carbohydrate and lipid metabolism and energy metabolism.
Li S et al. (2020) [71]	Cross-sectional study	↑ *Collinsella*, *Lactobacillus*, and *Succinivibrio ↓ Adlercreutzia*, *Anaerostipes*, and *Ruminococcus*	The abundance of *Succinivibrio* showed a positive correlation with both the total PANSS scores and the general PANSS scores, whereas *Corynebacterium* abundance was negatively associated with the PANSS negative scores .
Schwarz et al. (2018) [67]	Longitudinal (12 months) study	↑ *Lactobacillus*, *Tropheryma*, *Halothiobacillus*, *Saccharophagus*, *Ochrobactrum*, *Deferribacter* and *Halorubrum. ↓ Anabaena*, *Nitrosospira* and *Gallionella*	Elevated levels of *Lactobacillus* group bacteria were observed in FEP subjects and showed significant correlations with symptom severity across multiple domains.
Shen Y et al. (2018) [66]	Cross-sectional study	↑ Phylum: *Proteobacteria* Genera: *Succinivibrio*, *Megasphaera*, *Collinsella*, *Clostridium*, *Klebsiella*, and *Methanobrevibacter ↓ Coprococcus*, *Roseburia*, *Blautia*	-

**Table 3:** The most important information contained in the review related to the pathophysiology and treatment of depression and the impact of gut microbiota.

Publication	Information
Bear, T.L.K. et al., 2020 [37]	-Mediterranean diet reduces the level of Proteobacteria, while increasing beneficial bacteria (e.g., Bifidobacteria, Clostridium cluster XVIa) -Oxidative stress is a major factor in the development of mood disorders
Madabushi, J.S. et al., 2023 [43]	-Probiotics may be an effective measure to treat mental health disorders
Dash, S. et al., 2015 [45]	-Patients often exhibit shifts in gut microbiota composition—e.g., reduced diversity, altered abundances in phyla such as Firmicutes and Bacteroidetes
Góralczyk-Bińkowska, A. et al., 2022 [46]	-Disrupted tryptophan metabolism produces neurotoxic kynurenine pathway products linked to depression. -Gut dysbiosis contributes to mood changes, stress response, and psychiatric disorders like depression and anxiety.
Cheung, S.G. et al., 2019 [47]	-Depressed individuals consistent decreases in genera such as *Faecalibacterium* and *Coprococcus*; increases in *Actinobacteria* and *Alistipes* reported. -Gut microbiota alterations are linked with major depressive disorder and may affect inflammation and neurotransmitter pathways. -Potential for microbiome-targeted therapies exists but requires further well-controlled trials.
McGuinness, A.J. et al., 2022 [48]	-In patients with depression, an increased abundance of bacteria such as *Alistipes*, *Escherichia*, *Lactobacillus*, *Veillonella*, *Enterococcus*, *Flavonifractor*, *Eggerthella*, *Streptococcus*, and *Parabacteroides* has been observed, along with a reduced presence of *Prevotella*, *Coprococcus*, *Faecalibacterium*, and *Ruminococcus*
Randeni, N.; Xu, B., 2025 [53]	-Gut microbes produce SCFAs and other molecules that lower inflammation and regulate the HPA axis. -Serotonin, unable to cross the blood–brain barrier, still regulates gut movement, immunity, and the enteric nervous system.

## Data Availability

No new data were created or analyzed in this study.
